# Pathogenic Detection by Metagenomic Next-Generation Sequencing in Osteoarticular Infections

**DOI:** 10.3389/fcimb.2020.00471

**Published:** 2020-09-17

**Authors:** Zi-da Huang, Zi-jie Zhang, Bin Yang, Wen-bo Li, Chong-jing Zhang, Xin-yu Fang, Chao-fan Zhang, Wen-ming Zhang, Jian-hua Lin

**Affiliations:** ^1^Department of Orthopaedic Surgery, The First Affiliated Hospital of Fujian Medical University, Fuzhou, China; ^2^Department of Laboratory Medicine, The First Affiliated Hospital of Fujian Medical University, Fuzhou, China

**Keywords:** metagenomic next-generation sequencing, osteoarticular infection, pathogen, diagnosis, culture

## Abstract

**Objectives:** To evaluate metagenomic next-generation sequencing (mNGS) as a diagnostic tool in detecting pathogens from osteoarticular infection (OAI) samples.

**Methods:** 130 samples of joint fluid, sonicate fluid, and tissue were prospectively collected from 92 patients with OAI. The performance of mNGS and microbiology culture was compared pairwise.

**Results:** The overall sensitivity of mNGS was 88.5% (115/130), significantly higher than that of microbiological culture, which had a sensitivity of 69.2% (90/130, *p* < 0.01). Sensitivity was significantly higher for joint fluid (mNGS: 86.7% vs. microbiology culture: 68.7%, *p* < 0.01) and sonicate fluid (mNGS: 100% vs. microbiology culture: 66.7%, *p* < 0.05) samples. mNGS detected 12 pathogenic strains undetected by microbiological culture. Additional pathogens detected by mNGS were *Coagulase-negative Staphylococci, Gram-negative Bacillus, Streptococci, Anaerobe, non-tuberculosis mycobacterium, MTCP* (*p* > 0.05), and *Mycoplasma* (OR = ∞, 95% confidence interval, 5.12–∞, *p* < 0.001). Additionally, sensitivity by mNGS was higher in antibiotic-treated samples compared to microbiological culture (89.7 vs. 61.5%, *p* < 0.01).

**Conclusions:** mNGS is a robust diagnostic tool for pathogenic detection in samples from OAI patients, compared to routine cultures. The mNGS technique is particularly valuable to diagnose pathogens that are difficult to be cultured, or to test samples from patients previously treated with antibiotics.

## Introduction

Osteoarticular infections (OAI), infections of the bone or joint, can be very serious. OAI might be difficult to be treated, which is associated with a high recurrence rate, long-term disability, and even mortality (Parvizi et al., 2014; Yombi et al., 2017). A prompt and accurate microbiological diagnosis facilitates the timely and precise application of antimicrobial therapy or surgery (Osmon et al., 2013). Conventional microbiology culture is the main diagnostic approach for most of the hospitals. Unfortunately, the incidence of culture-positive infection is reported to be 40–70% (Trampuz et al., 2007), affected by prior antibiotic usage, fastidious pathogens, and biofilm adhered to surface of implant (Tzeng et al., 2015). Furthermore, it always takes 2–14 days and even 6 weeks to culture and subsequently identify the microorganisms. Thus, there are several limitations associated with this technique.

Recently, molecular diagnostics have been applied to the diagnosis of OAI. These techniques encompass culture-independent approaches that directly detect the nucleic acid in clinical specimens within hours of running the assay (Levy et al., 2013). 16S rRNA/rDNA gene broad-range PCR is a universally accepted method for pathogen identification. However, because of the incapability of identifying fungal and polymicrobial infections, the sensitivity was not high for around 70% (Huang et al., 2018). Multiplex PCR can detect several specific pathogens in an assay (Malandain et al., 2018), but the number of targets is limited (<20). Microarray and PCR-based electron spray mass spectrometry are able to screen more microorganisms; however, the targeted probes or primers are pre-set and difficult to be updated (Peel et al., 2015).

Metagenomic next-generation sequencing (mNGS) is an agnostic approach for theoretical identification of all genome-known microorganisms from clinical specimen by high-throughput sequencing and automated bioinformatic analysis within a short turnaround time (1–6 days). mNGS has been used to detect pathogens from cerebral spinal fluid (Guan et al., 2016), blood (Grumaz et al., 2016), urine (Mouraviev and McDonald, 2018), and bronchoalveolar lavage fluid samples (Miao et al., 2018). Some studies reported significant improvement (sensitivity 88% and specificity 89%) of detection of pathogens by using the mNGS method in patients with prosthetic joint infection (PJI) (Street et al., 2017). However, research so far has focused on a specific type of sample, like sonication fluid or synovial fluid. There have been few cohort studies reported that investigate the capability of mNGS in detecting pathogens from different types of samples in OAI patients. On the other hand, mNGS results are highly associated with the sequencing platform, bioinformatic pipeline, and environment of lab, contributing to the complexity of interpreting mNGS results (Schlaberg et al., 2017). More investigations from separate institutions are needed to evaluate the diagnostic performance of mNGS.

In our study, we assessed the performance of mNGS in universally detecting pathogens from various types of OAI samples in real-world clinical practice.

## Patients and Methods

### Study Population

The present study initially included 142 osteoarticular samples from 103 patients undergoing conservative or surgical therapy for OAI. The physicians and orthopedic surgeons diagnosed patients with infectious diseases according to the clinical manifestation, laboratory tests, imaging, and histopathology examinations. The data were prospectively collected and analyzed at a single institution, which is the local quality control center of joint arthroplasty (the First Affiliated Hospital of Fujian Medical University, Fuzhou, China), over a span of 12 months, from December 2017 to December 2018. Twelve samples from 11 patients were excluded due to various reasons, such as obvious contamination during sampling or transportation, failed library conduction or sequencing, inadequate volume of sample for culture and molecular tests, and samples collected from children. In addition, a total of five joint fluid and five synovial tissue samples from five patients with primary total knee arthroplasty due to osteoarthritis were obtained as derivation samples previously in order to determine the potential background microbes in laboratory and joint cavity.

### Definitions

All cases included in our study fulfilled the main diagnostic criteria for each type of OAI. According to our protocol, we considered patients with a history of a warm, swollen, and tender joint to have primary septic arthritis (PSA) and arthrocentesis would be performed to obtain synovial fluid samples for microbiological analyses. Meanwhile, patients with clinical infectious symptoms of bones, radiological study that identified the location of the infection, and a pair of positive blood or bone-sample cultures were confirmed as osteomyelitis (OM). Prosthetic joint infection (PJI) was defined as the isolation of a pathogenic microorganism from two or more surgical, joint-aspirated or blood cultures, or by one such positive culture plus the presence of typical signs and clinical symptoms after joint arthroplasty (inflammatory signs, the presence of a sinus tract, or purulence around the prosthesis during surgery). Implant-associated infection (IAI) was diagnosed when growth of a microorganism in at least two intraoperative peri-implant tissue samples or sonication fluid of the removed implant was documented. In general, our center is concentrated on improving the diagnosis of PJI, so we consider it as a specific classification of OAI, apart from normal IAI. Surgical site infection is defined by the following criteria: (1) infection arising within a short history (30–90 days) of the index surgery (where day 1 is the procedure date), (2) involving tissues related to the incision (e.g., fascial and muscle layers, skin or subcutaneous), and (3) purulent drainage from incision or organisms identified from aseptically obtained specimen from incision by a culture-based microbiologic testing method performed for the purposes of clinical diagnosis or treatment.

### Clinical Sample Collection and Processing

Joint fluid was collected by needle aspiration preoperatively or intraoperatively. Sonication fluid was obtained from removed prostheses or components, following a previously described procedure. Tissue specimens were collected in the most inflammatory site during surgery, avoiding necrotic tissues. After sample collection, they were sent to the microbiology lab in the same institution where the samples were collected. For conventional microbiology cultures, samples were stored at room temperature for up to 30 min. For mNGS tests, samples were stored at −20°C and sent to the molecular lab of BGI company (Shenzhen, China) within 24 h. Each sample was divided equally to reduce potential heterogeneity and processed for culture and mNGS in a pairwise way.

### Conventional Microbiology Culture

Joint fluid and sonication fluid were inoculated in 0.1-ml aliquots onto aerobic blood agar and anaerobic blood agar. Next, they were incubated at 35–37°C in 5–7% CO_2_ aerobically and anaerobically for 6 and 14 days. Residual synovial-fluid volumes of >1 ml were injected into a BACTEC Peds Plus/F bottle and incubated in a BACTEC 9050 instrument (Becton-Dickinson GmbH, Heidelberg, Germany) for 6 days and subcultured if positive. Tissue was homogenized with broth and inoculated onto sheep blood agar, cultured aerobically and anaerobically as fluid samples. Samples were incubated onto a modified Loewenstein–Jensen medium at 37°C for 42 days, when patients were highly suspected to have tuberculosis infection. Any growth from joint fluid was considered positive. Meanwhile, a tissue culture was regarded as positive when the same organism was isolated from ?2 samples. Growth of ≥20 colony-forming units/plate in sonication fluid samples was considered positive (Trampuz et al., 2007). All bacteria or fungi isolated were identified using the Vitek 2 system (bioMérieux Vitek, Inc., Hazelwood, Missouri, USA).

### Metagenomic Next-Generation Sequencing and Analysis

#### DNA Extraction

After vortexing for 5 min with 1 g of 0.5-mm glass beads to break the cell wall, DNA were extracted from the fluid specimens in 0.5-ml aliquots, or tissues in 25 mg volume with TIANamp Micro DNA Kit (DP316, TIANGEN Biotech, Beijing, China) following the manufacturer's protocol.

#### Library Conduction and Sequencing

The extracted DNA was sonicated to generate 200–300-bp fragments. Libraries were constructed through amplification by PCR and generation of single-stranded circles by circularization reactions. The quantified libraries were processed for 50-bp single-end sequencing on the BGISEQ-500 platform (BGI-Wuhan, Wuhan, China). Samples were proceeded in batches, with a negative control (whole-blood sample, proved by previous isolated mNGS analysis) taken from healthy donors prepared alongside each batch to monitor contamination. If suspected contamination was detected from sequencing results of negative control, the involved batch was reprocessed from the extraction step.

#### Bioinformatic Pipeline

Raw sequencing data was analyzed by a bioinformatic pipeline developed by BGI (Cai et al., 2020), which included the following steps. (1) Short (length <35 bp) and low-quality reads were filtered. (2) Human host sequences were eliminated by mapping to the human reference genome (hg19) using BWA (Burrows-Wheeler alignment, http://bio-bwa.sourceforge.net). (3) After removal of low-complexity reads, the remaining sequencing data was simultaneously aligned by BWA to 4 microbial genome databases, consisting of viruses, bacteria, fungi, and parasites, to generate an original mapped list. (4) The in-house built reference database contained 2,700 viruses, 1,494 bacterial species, 73 fungal species, 48 parasites, and 40 mycoplasma/chlamydia, all related to human diseases. The reference genomes in the database were downloaded from the National Center for Biotechnology Information (ftp://ftp.ncbi.nlm.nih.gov/genomes/).

### Result Interpretation

The number of total raw reads varied among different samples. In order to reach an equalized comparison, the standardized ratio (SR) was defined as number of total reads/20,000,000. The stringently mapped read number in genus level (SMRNG) was standardized as SDSMRNG via multiplication by SR, and the stringently mapped read number in species level (SMRN) was standardized as SDSMRN via multiplication by SR. The coverage rate was defined as number of matched reads ×50/genome length. Optimal thresholds were set up as below in order to identify true pathogens using previously studied data (Cai et al., 2020).

1. SDSMRNG <3 was considered as insignificant, except for *Mycobacterium tuberculosis*. *M. tuberculosis* was considered positive when ≥1 read was mapped at the genus level as *M. tuberculosis* complex (MTCP), due to its low DNA yield and possibility for contamination.
2. *Burkholderia, Ralstonia, Cupriavidus, Acidovorax*, and *Delftia* were considered as positive when relative abundance for each genus level ≥85%, since they were regarded as the most common contamination genera in the lab and were rarely cultured as pathogens of OAI.
3. The relative abundance in non-human genus level ≥15% were determined as the optimal threshold for bacterial identification, while relative abundance at the genus level ≥30% for fungi.
4. Microbial species whose coverage rate or relative abundance at the species level was of the first rank within positive genus and with SDSMRN ≥3 were determined as positive.

### Statistical Analysis

A comparative analysis was conducted using the Pearson χ^2^ test, McNemar test, or Fisher exact test when appropriate. Data analyses were performed using the SPSS v21.0 statistical software package (SPSS Inc., Chicago, Illinois, USA).

## Results

### Sample Characteristics

**Figure 1:**
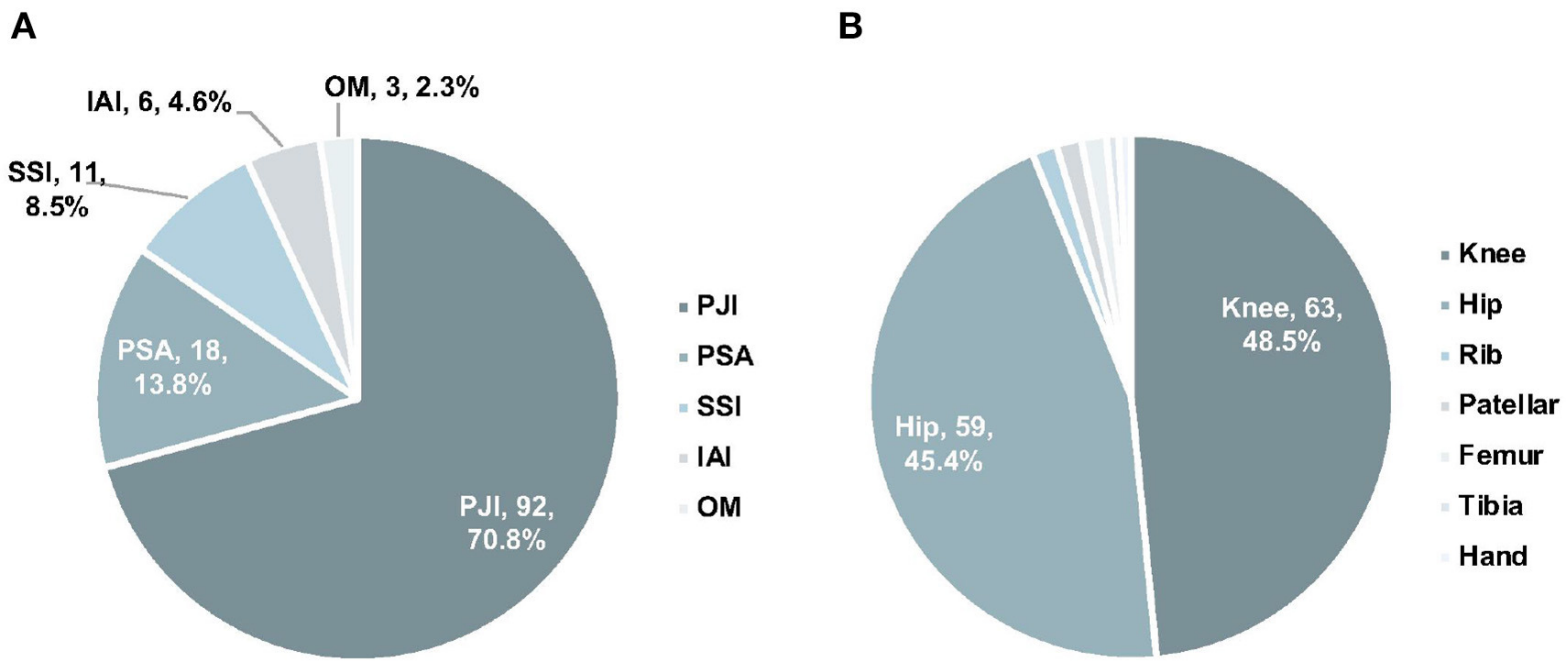
The distribution of infection types **(A)** and anatomic collection sites **(B)**. PJI, prosthetic joint infection; IAI, implant-associated infection; SSI, surgical site infection; PSA, primary septic arthritis; MTCP, *mycobacterium tuberculosis* complex.

Among the 130 samples from 92 OAI patients, 84 were joint fluid, 24 were sonication fluid, and 23 were tissues. Sources of samples included 63 knees, 59 hips, 2 ribs, 2 patellars, 2 femurs, 1 tibia, and 1 hand (Figure 1A). Ninety-two samples were collected from PJI, while 18 from PSA, 6 from IAI, 11 from SSI, and 3 from OM (Figure 1B). Clinical review data were listed in Supplementary Table 1.

### mNGS Sequencing Information

**Figure 2:**
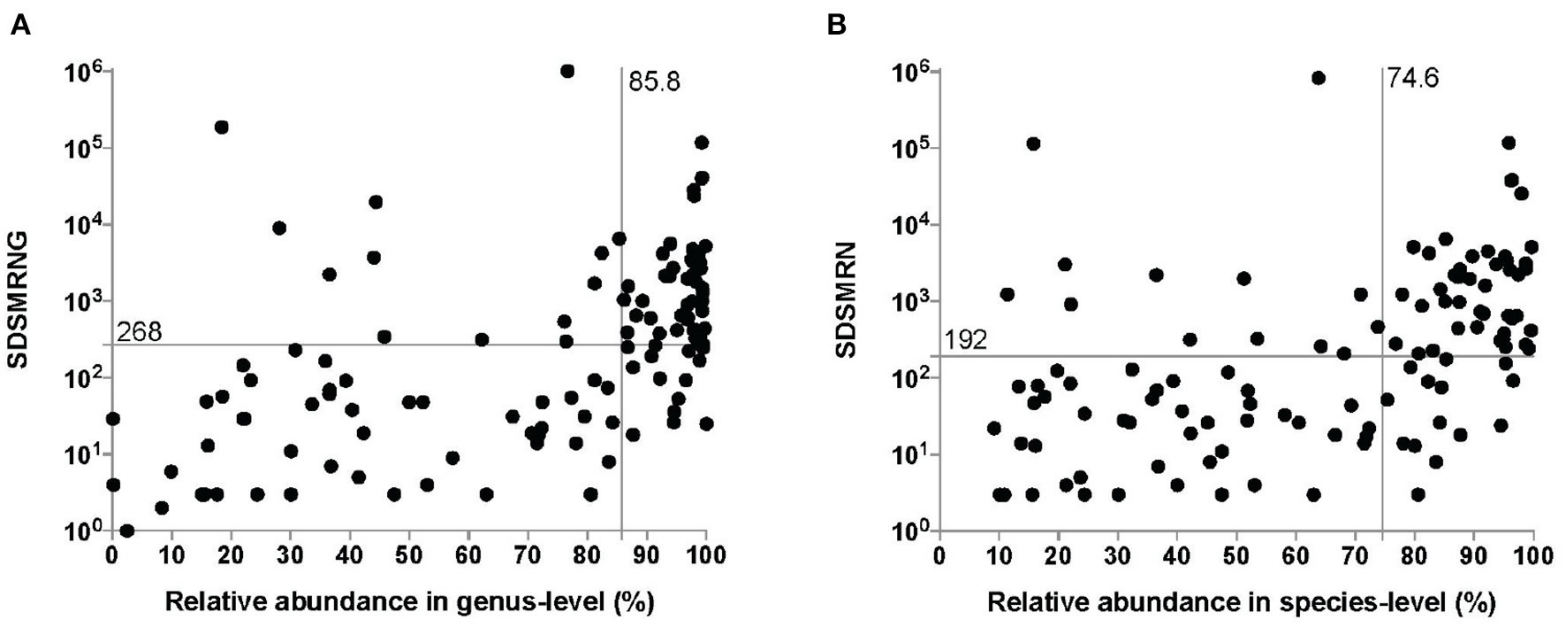
The number of unique reads and relative abundance of identified pathogens by the metagenomic next-generation sequencing (mNGS) approach at the genus level **(A)** and species level **(B)**. The median number of unique reads and the relative abundance at the genus level were 268 and 85.8%, respectively. The median number of unique reads and the relative abundance at the genus level were 192 and 74.6%, respectively. SDSMRNG, standardized number of reads stringently mapped to pathogen in genus level; SDSMRN, standardized number of reads stringently mapped to pathogen in species level.

It took between 26 and 48 h for the entire procedure of mNGS analysis to generate an interpretable result. An average of 21,142,755 total reads (range: 5,308,500–57,552,330 reads) were generated from sequencing. 124 pathogens were identified at the genus level in the interpreted results of mNGS (Figure 2A), with an average SDSMRNG of 268 (range, 1–1,021,637 reads) and an average relative abundance at the genus level of 85.8% (range 0.1–100%). There were 116 pathogens identified at the species level (Figure 2B), with an average SDSMRN of 192 (range 3–830,878 reads) and an average of relative abundance at the species level as 74.6% (range 9.2–99.7%). Relative abundance of host reads was at an average of 96.83% (range 93.55–99.23%). No pathogens other than common contaminants detected in the laboratory as those encountered in previous studies (Salter et al., 2014) were identified either in the derivation samples or in the negative-control samples. Bacterial and fungal classification results from OAI samples are listed in Supplementary Tables 2, 3, while results in derivation ones were in Supplementary Tables 4, 5.

### Concordance Between the mNGS and Microbiological Culture Methodologies

**Figure 3:**
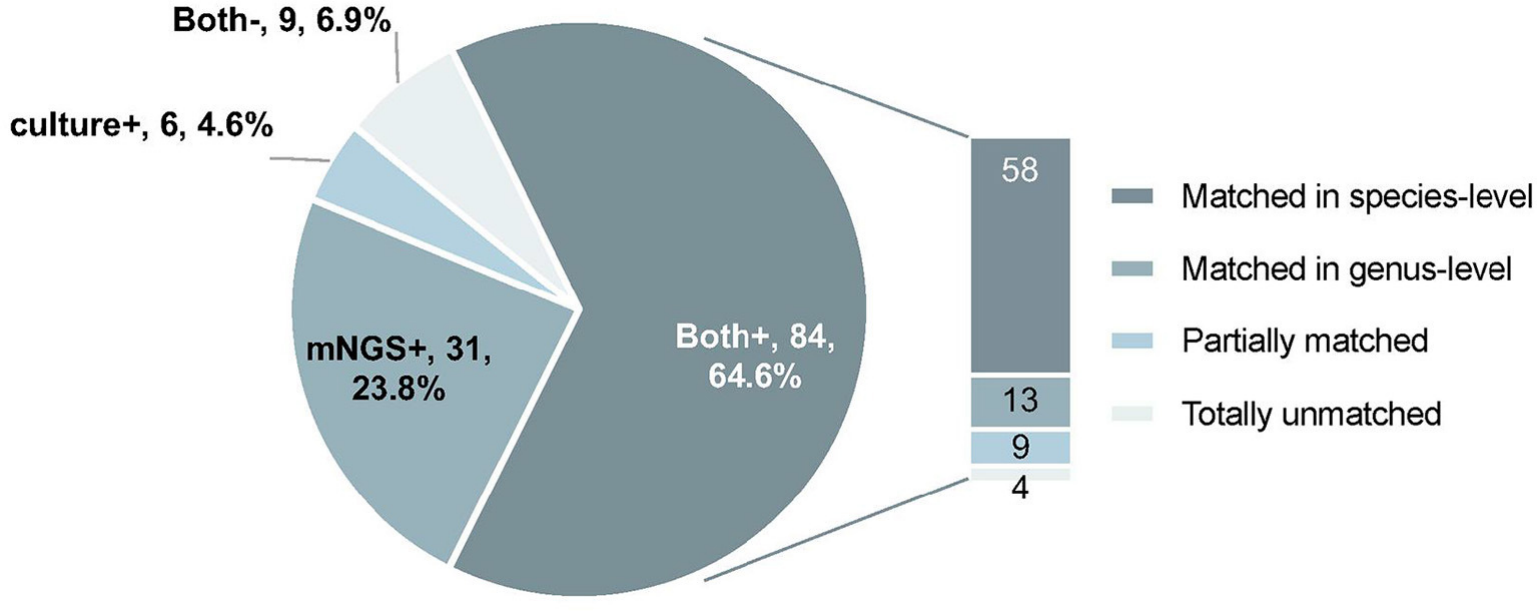
The concordance of results between metagenomic next-generation sequencing (mNGS) and microbiological culture. Both+, results of mNGS and culture were both positive; Both–, results of mNGS and culture were both negative; mNGS+, only the mNGS result was positive, culture was not; Culture+, only the culture result was positive, mNGS was not.

mNGS and microbiological cultures were both positive in 58 of 130 (44.6%) samples and were both negative in 9 of 130 (6.9%) samples. Thirty-one samples were positive by mNGS only (23.8%), and 6 were positive by culture only (4.6%). For the samples where mNGS and culture were both positive, 58 of 84 (69.0%) were totally matched at the species level. For the same set of samples, 13 of 84 (15.5%) were matched at the genus level. Of these, 9 of 84 (10.7%) were regarded as partially matched, which means at least one sample pathogen was detected in polymicrobial results, and 4 of 84 (4.8%) were totally unmatched (Figure 3).

mNGS could detect 12 more potential pathogens from samples where mNGS and culture were both positive and 33 more potential pathogens from samples where culture were negative. We also found a number of negative cultures. These include the presence of fastidious microorganisms that may need special culture condition (e.g., *Anaerobes, Mycobacterium, Mycoplasma*), prior use of antibiotics, misclassification by mNGS, and inhibition by other organisms in polymicrobial infections (Table 1). However, 16 culturable pathogens were also missed by mNGS because of the low read numbers, low relative abundance, a contaminated culture, and strains being unidentifiable by mNGS (Table 2).

**Table 1:** Analysis of culture-negative pathogens detected by metagenomic next-generation sequencing.

**Pathogens**	**No.**	**Possible culture-negative reasons**				
		**Prior use of antibiotics**	**Fastidious organisms**	**Polymicrobial**	**Misclassification**	**Unknown**
*Gram-negative bacillus*	9	7	0	2	1	0
*Anaerobe*	8	2	8	0	0	0
*Mycoplasma*	8	0	8	0	0	0
*Coagulase-negative Staphylococci*	7	5	0	0	0	2
*Mycobacterium*	7	2	7	0	0	0
*Fungi*	3	0	0	0	0	3
*Staphylococcus aureus*	2	2	0	0	0	0
*Streptococci*	2	2	0	0	0	0
*Corynebacterium* spp.	1	0	0	0	0	1

**Table 2:** Analysis of culturable pathogens missed by metagenomic next-generation sequencing (mNGS).

**Patient No**.	**Sample No**.	**Diagnosis**	**Sample type**	**mNGS results**	**Culture results**	**mNGS missed pathogens**	**Possible explanations**
19	17S0146542	PJI	Joint fluid	*Candida parapsilosis* *Cutibacterium acnes*	*Staphylococcus epidermidis* *C. parapsilosis*	*S. epidermidis*	Low read number
32	17S0284314	PJI	Joint fluid	*Mycoplasma hominis*	*Candida tropicalis*	*C. tropicalis*	Not detected
32	17S0284313	PJI	Tissue	*Mycoplasma hominis*	*Candida tropicalis*	*C. tropicalis*	Not detected
32	18S0184696	PJI	Joint fluid	*Mycoplasma hominis*	*Candida tropicalis*	*C. tropicalis*	Not detected
49	18S0184868	PJI	Joint fluid	Negative	*S. epidermidis*	*S. epidermidis*	Not detected
50	18S0184897	PJI	Joint fluid	*Enterococcus faecalis* MTCP	*E. faecalis* *S. epidermidis* *A. baumannii*	*Acinetobacter baumannii* *S. epidermidis*	Low relative abundance Low relative abundance
50	18S0184898	PJI	Sonication fluid	*E. faecalis* MTCP	*E. faecalis* *S. epidermidis baumannii* *Mycobacterium abscessus*	*baumannii* *S. epidermidis*	Low relative abundance Low relative abundance
73	18S4338147	SSI	Tissue	*Candida albicans*	*C. albicans* *E. faecalis*	*E. faecalis*	Not detected
77	18S4338151	PJI	Tissue	Negative	*Staphylococcus saprophyticus*	*S. saprophyticus*	Not detected
103	18S4005284	PJI	Joint fluid	Negative	*E. faecalis*	*E. faecalis*	Not detected
114	18S4005259	SSI	Joint fluid	Negative	*M. abscessus*	*M. abscessus*	Low reads number
121	18S3301078	IAI	Tissue	*Klebsiella pneumoniae* *Morganella morganii*	*E. faecalis* *E. coli*	*E. faecalis* *E. coli*	Low relative abundance Low relative abundance
123	18S3301116	PSA	Joint fluid	*Prevotella intermedia*	*A. baumannii*	*A. baumannii*	Not detected

*PJI, prosthetic joint infection; IAI, implant-associated infection; SSI, surgical site infection; PSA, primary septic arthritis; MTCP, mycobacterium tuberculosis complex*.

### Comparison of Pathogenic Detection Between mNGS and Culture Testing

**Figure 4:**
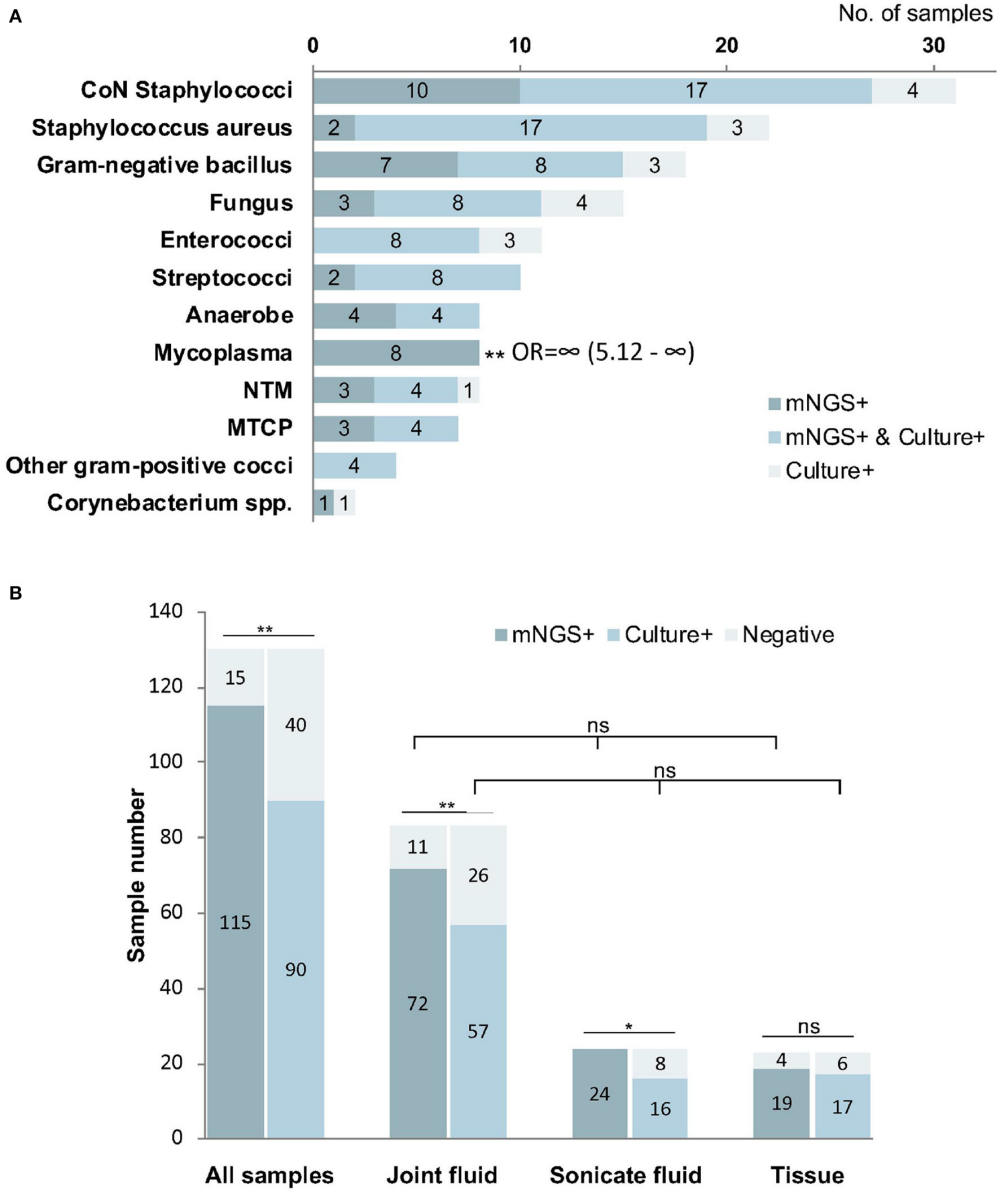
The positivity of metagenomic next-generation sequencing (mNGS) and culture for different pathogens **(A)** and sample types **(B)**. Mycoplasma were more frequently identified by mNGS than culture (*P* < 0.01). The overall positive rate of mNGS is significantly higher than that of culture (*P* < 0.01), especially from joint fluid (*P* < 0.01) and sonicate fluid (*P* < 0.05). *P < 0.05; **P < 0.01; mNGS+ and culture+, the results of mNGS and culture were both positive; mNGS+, only the mNGS result was positive, culture was not; Culture+, only the culture result was positive, mNGS was not.

Among the 144 pathogens detected by mNGS or culture, *Coagulase-negative Staphylococci* (*n* = 31) was the most commonly detected genus, followed by *Staphylococcus aureus* (*n* = 22), *Gram-negative bacillus* (*n* = 18), *Fungus* (*n* = 15), *Enterococci* (*n* = 11), and *Streptococci* (*n* = 10). Although mNGS showed higher positive rates of detection than culture in *coagulase-negative Staphylococci, Gram-negative bacillus, Streptococci, Anaerobe, non-tuberculosis mycobacterium*, and *MTCP*, the only significant difference was in *Mycoplasma* (*p* < 0.001). However, the positive rate of mNGS was lower than that in culture for *Enterococci, Fungus*, and *Staphylococcus aureus* detection, with no significant differences (Figure 4A).

### Comparison of mNGS and Culture Testing Among Different Samples

Among all types of samples, the positive rate of mNGS (88.5%) was significantly higher than that in culture (69.2%, *p* < 0.01). We found that the percentage of mNGS-positive cases was significantly higher than that of culture-positive cases in joint fluid (86.7 vs. 68.7%, *p* < 0.01) and sonication fluid (100 vs. 66.7%, *p* < 0.05). However, the difference in tissue samples was not significant between mNGS and culture (*P* > 0.05). The differences in the positive rate were not significant among joint fluid, sonication fluid, and tissue by either mNGS or culture methods (*p* > 0.05, Figure 4B).

### Effect of Prior Antimicrobial Therapy on Pathogen Detection

**Figure 5:**
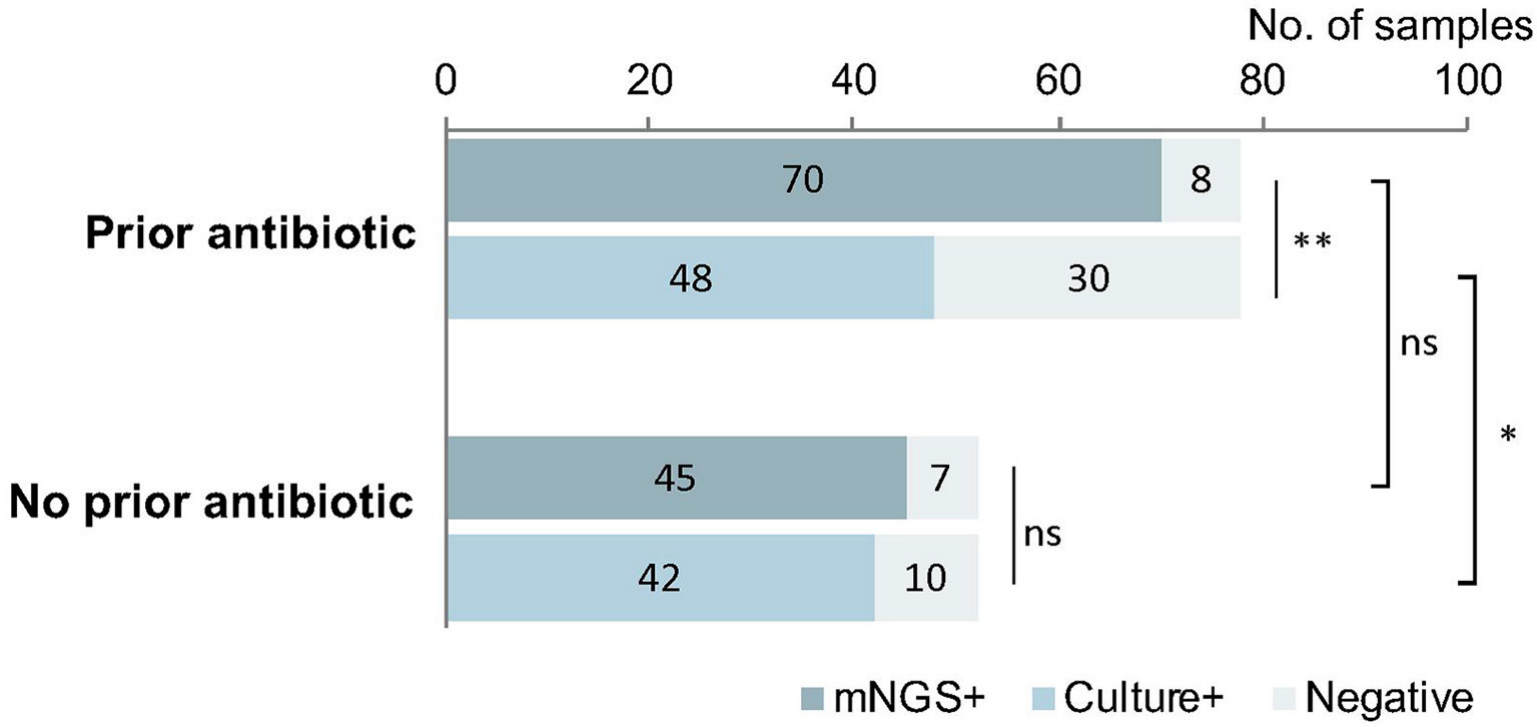
The implication of prior antimicrobial therapy on pathogen detection. mNGS could detect the pathogens from 89.7% samples previously treated with antibiotic, much higher than the cultured samples previously treated with antibiotic (61.5%, *p* < 0.01). Meanwhile, among the samples without prior use of antibiotic, the difference in the detection by mNGS vs. culture was not significant (86.5 vs. 80.8%; *p* > 0.05). The positive rate of mNGS between the groups with and without antibiotic were similar (*P* > 0.05), but the positive rate in the group with antibiotic was much lower than that in the group without antibiotic (*p* < 0.05). *p < 0.05; **p < 0.01.

In the 78 samples that received antimicrobial therapy prior to sampling, mNGS detected pathogens from 70 (89.7%) samples. The detection rate of mNGS was greater than that of culture (61.5%, *p* < 0.01). Meanwhile, there was no difference in the positive detection rate between mNGS and culture for the 52 samples that were not exposed to antibiotic prior to sampling (mNGS: 86.5% vs. culture: 80.8; *p* > 0.05). These data are summarized in Figure 5.

## Discussion

In the present study, the performances of mNGS and culture in detecting pathogens from a variety of sample types in OAI patients were systematically compared. The mNGS methodology showed several advantages. Firstly, the overall detection ratio of pathogens by mNGS was significantly higher than that by conventional culture, especially in the samples from joint fluid and sonication fluid. Due to the higher sensitivity, these samples might be optimal candidates for mNGS testing. Secondly, mNGS was less affected by antimicrobial therapy. The result was consistent with prior literature regarding molecular techniques (Vasoo et al., 2015), indicating that the mNGS was sensitive enough to detect low levels of microorganisms or residual nucleic acid in specimens. Additionally, mNGS was found to be capable of detecting more potential pathogens, which were missed by culture in those culture-positive samples (Ivy et al., 2018), facilitating identification of underestimated polymicrobial OAI, which might lead to a higher recurrence rate (Calvo et al., 2016). Moreover, it takes only 24–48 h for the present mNGS methods to generate a final report after receiving the specimen. This time frame is not only much faster than the current culture workflow (2–14 days) but also faster than most mNGS procedures published (24 h to 6 days) (Simner et al., 2018). The mNGS method could provide prompt guidance for targeted antimicrobial treatment during emergency surgery in real-world clinical practice.

The mNGS methodology and analysis were shown to be more accurate in detecting *Mycoplasma* (*p* < 0.01) compared to microbial culture methods. *Coagulase-negative staphylococci, Gram-negative bacillus, Streptococci, Anaerobe, non-tuberculosis mycobacterium*, and *MTCP* detection by mNGS did not show a significant difference in comparison to culture. The possible reasons for the false-negative cultures are numerous and complex. Besides antibiotics, the growth of less abundant strains could be affected by inhibition of coexistent microorganisms. Moreover, microorganisms requiring special cultivation condition have been shown to be commonly missed by regular culture (Juchler et al., 2018). mNGS overcomes the shortcomings of culture by direct sequencing from clinical specimens. However, misclassification caused by the short length of sequencing read, incomplete databases, improper algorithms, or highly homologous genomes will produce false-positive results (McIntyre et al., 2017) [e.g., *Escherichia coli* was often misaligned as *Shigella dysenteriae* due to high similarity between the two genomes (Chattaway et al., 2017)] or discordant results (e.g., *Staphylococcus aureus* was occasionally aligned as *Staphylococcus epidermidis*, if the coverage rate is too low to be distinguished precisely).

Although the average total read number generated (around 20,000,000) was similar to other reports, the numbers of reads mapped to pathogens were much lower in our study. The low yield of mapped reads may cause false-negative mNGS results, even in those culturable pathogens. This may be due to the absence of an enrichment procedure for bacterial DNA by depletion of host nucleic acids (Thoendel et al., 2016). The removal of human DNA by using the lysate of the cell membrane will potentially remove the DNA of the virus, parasite, or bacteria with a fragile cell wall as well. However, the workflow we utilized was developed to be a universal procedure, which includes virus and parasite (rare causative organisms of OAI) besides bacteria and fungus. A customized method might address this problem.

Another aspect that might complicate the interpretation of the mNGS result is the background signal (Bukowska-Ośko et al., 2016), contributed by transient colonizers, potential microbiota (Jakobsen et al., 2018), or contamination from reagents (Thoendel et al., 2017), lab environment, or the air. Although mNGS takes a further step than PCR in quantifying pathogen reads absolutely or as percentage of the total number of sequenced reads, it is still a challenge in setting up an optimal threshold to distinguish real pathogens from background organisms. Especially for low-grade infection caused by low-abundance pathogens, the overwhelming background noise is a confounding factor. On the other hand, among polymicrobial infections, the relative abundance of minority pathogens might be far lower than predominant pathogens and accurately filtered out by the threshold accordingly (as indicated in Table 2). Comparing the result with the negative control without template (Wilson et al., 2014) was futile in our setting, since the absence of human DNA in controls led to a biased amplification and sequencing of microorganisms (unpublished data). The introduction of an extra “spike-in” calibration (Stämmler et al., 2016) might act as a solution in the near future.

Although we have demonstrated success in pathogenic detection in OAI patients' samples using the mNGS (Huang et al., 2019), this study has its limitations. This study was performed at a referral center for OAI patients. Therefore, the samples containing rare pathogens or non-implant-associated infections were limited, which might not reflect the real performance of mNGS in clinically relevant situations. In addition, considering the balance between cost and efficiency, the depth of mNGS is not as deep as whole-genome sequencing, which might diminish the possibility of generating adequate reads of pathogens. Furthermore, lack of non-infection samples as a matched control group diminished the diagnostic value of OAI by mNGS vs. culture. Because it is difficult to set a perfect control group for the diversity of infection type and source. Finally, the antibiotic resistance gene could not be detected by the present mNGS approach due to the inadequate coverage rate of reference genome caused by low yield of pathogen-mapped reads.

In conclusion, direct analysis of joint fluid and sonication fluid from OAI patients with mNGS is complementary to routine cultures in rapidly identifying more pathogens, which are difficult to be cultured or detected from patients previously treated with antibiotics.

## Data Availability Statement

The data that support our finding of this study have been deposited in CNSA (http://db.cngb.org/cnsa/) of China National GeneBank Database (CNGBdb) with accession number CNP0001047.

## Ethics Statement

The studies involving human participants were reviewed and approved by the guidelines set forth by First Affiliated Hospital of Fujian Medical University Institutional Review Board (Protocol # 2017-094 and 2018-026). The patients/participants provided written informed consent to participate in this study.

## Author Contributions

ZZ and ZH conceived and designed the study. WL, ZH, and BY analyzed the data. C-jZ collected the related clinical information. ZZ, ZH, WL, C-jZ, XF, and C-fZ conducted the infection samples associated with the study. BY provided the technical support. ZZ and ZH wrote the draft, JL and WZ revised it. All authors approved the final version. All authors contributed to the article and approved the submitted version.

## Conflict of Interest

The authors declare that the research was conducted in the absence of any commercial or financial relationships that could be construed as a potential conflict of interest.
